# Supplementary material for: Type II taste cells participate in mucosal immune surveillance
Source: PLoS Biol. 2023 Jan 12;21(1):e3001647. doi: 10.1371/journal.pbio.3001647 (PMC9836272; doi:10.1371/journal.pbio.3001647)
Supplement: S1 Code — The code for analysis of gustometer data in Fig 4 for sucrose is shown below. The codes for other tastants were similar except for corresponding changes in file names and concentrations. (DOCX) [file pbio.3001647.s002.docx]

require(tidyverse)

require(emmeans)

Sucrose<-read_delim("sucrose1.txt") %>%
 mutate(Concentration=factor(Concentration,levels=c("30 mM","100 mM","300 mM","1000 mM")))

Sucrose.lm<-lm(LickRatio~Genotype*Concentration,data=Sucrose)
joint_tests(Sucrose.lm)

(Sucrose.emm<-emmeans(Sucrose.lm,~Genotype*Concentration))

contrast(Sucrose.emm,"pairwise",simple="each",adjust="tukey")
