# Supplementary figures and images for: Type II taste cells participate in mucosal immune surveillance
Source: PLoS Biol. 2023 Jan 12;21(1):e3001647. doi: 10.1371/journal.pbio.3001647 (PMC9836272; doi:10.1371/journal.pbio.3001647)

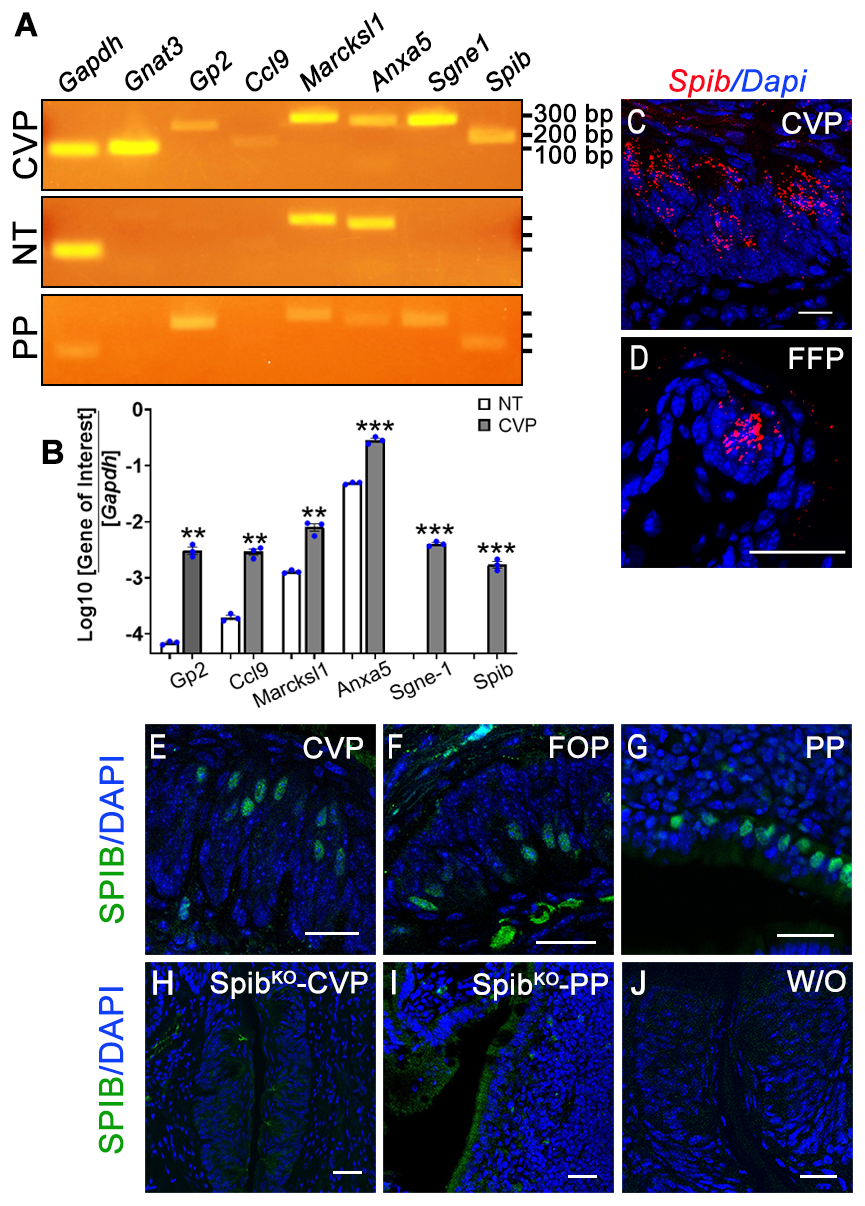

Supplement: S1 Fig — (A) End point PCR (35 cycles) of Gapdh (housekeeping control gene) Gnat3 (taste tissue control gene), Gp2, Ccl9, Marcksl1, Anxa5, Sgne1, and Spib from cDNA prepared CVP, non-taste lingual epithelium (NT), and Peyer’s Patch (PP). Gp2, Ccl9, Sgne1, and Spib are expressed in CVP and PP, but not in NT. Marcksl1 and Anxa5 are expressed in all tissues, although at lower levels in the NT. Lines on the right indicate position of molecular weight markers. (B) qPCR analysis of above genes expression in CVP and NT confirms that all M cell marker genes are highly expressed in CVP, while they are expressed at lower levels or not at all in NT. The expression of each marker gene is plotted as the logarithm of the ratio between its cycle threshold values to those of Gapdh. Individual datapoints from each cDNA sample in shown in blue dots (the underlying data can be found in Data B in S1_Data). (C-D) RNAscope hybridization using an Spib-specific probe set produced strong signals in subsets of taste cells in CVP and FFP. (E-G) Indirect immunofluorescence confocal microscopy of cryosection from taste papillae and PP stained with an SPIB antibody shows strong nuclear staining in subpopulations of taste cells in CVP, FOP, and PP. (H-I) Absence of SPIB signal in CVP and PP of SpibKO mice proves the specificity of SPIB antibody. (J) Omission of the primary antibody (W/O) demonstrates low nonspecific background from secondary antibody in CVP. Nuclei are counter stained with DAPI in panels C-J. Scale bars, 50 μm. **p < .01, ***p < .001. (TIF) [file pbio.3001647.s003.tif]

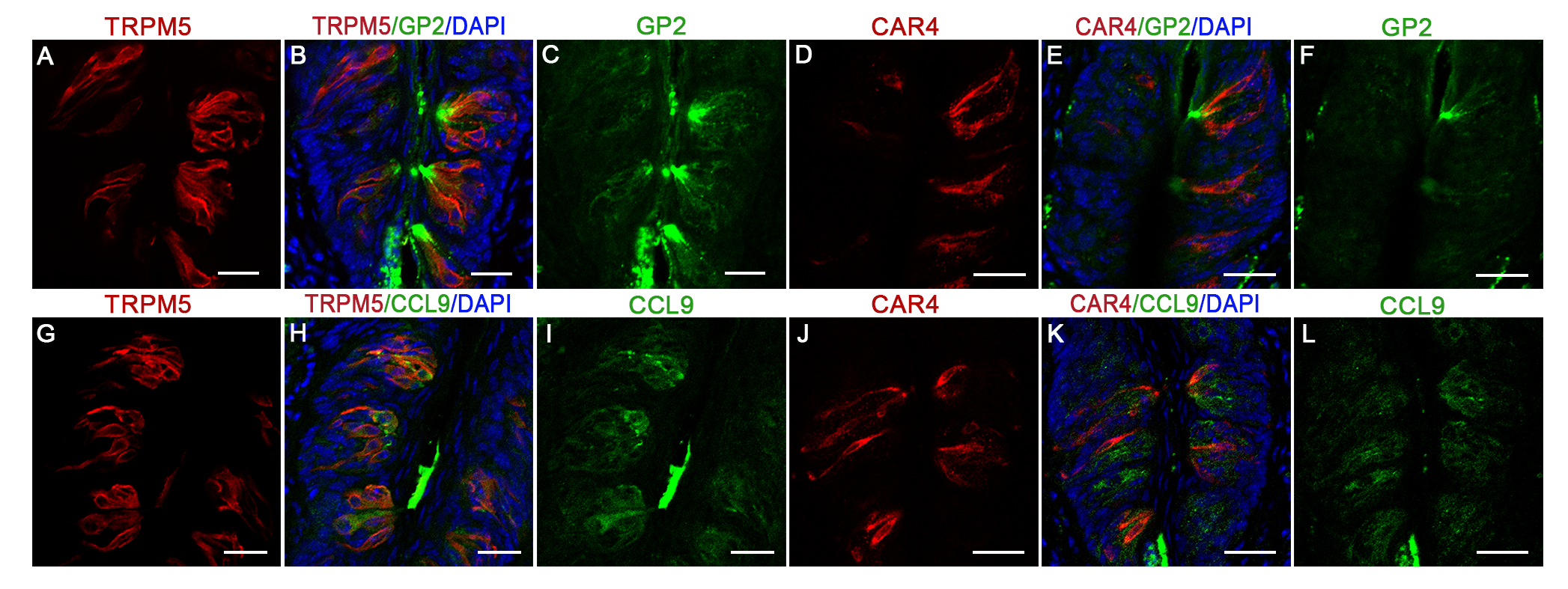

Supplement: S2 Fig — Double-labeled immunofluorescence confocal microscopy of CVP sections with antibodies against M cell markers GP2 and CCL9, with the type II taste cell marker TRPM5 (A-C and G-I) or the type III taste receptor marker CAR4 (D-F and J-L) in the CVP. Merged images show GP2 and CCL9 are coexpressed with TRPM5 (B, H), but not CAR4 (E, K). Nuclei are counterstained blue with DAPI. Scale bar, 50 μm. (TIF) [file pbio.3001647.s004.tif]

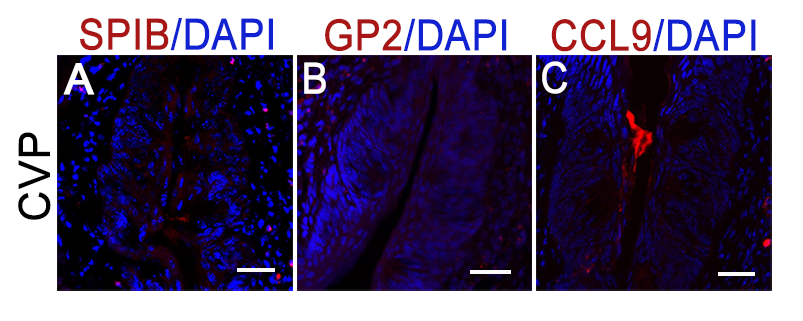

Supplement: S3 Fig — Immunofluorescence images with antibodies against SPIB (A), GP2 (B), and CCL9 (C) show no staining in CVP from Pou2f3 knockout mice that lack all type II taste cells. Nuclei are counterstained blue with DAPI. Scale bar, 50 μm. (TIF) [file pbio.3001647.s005.tif]

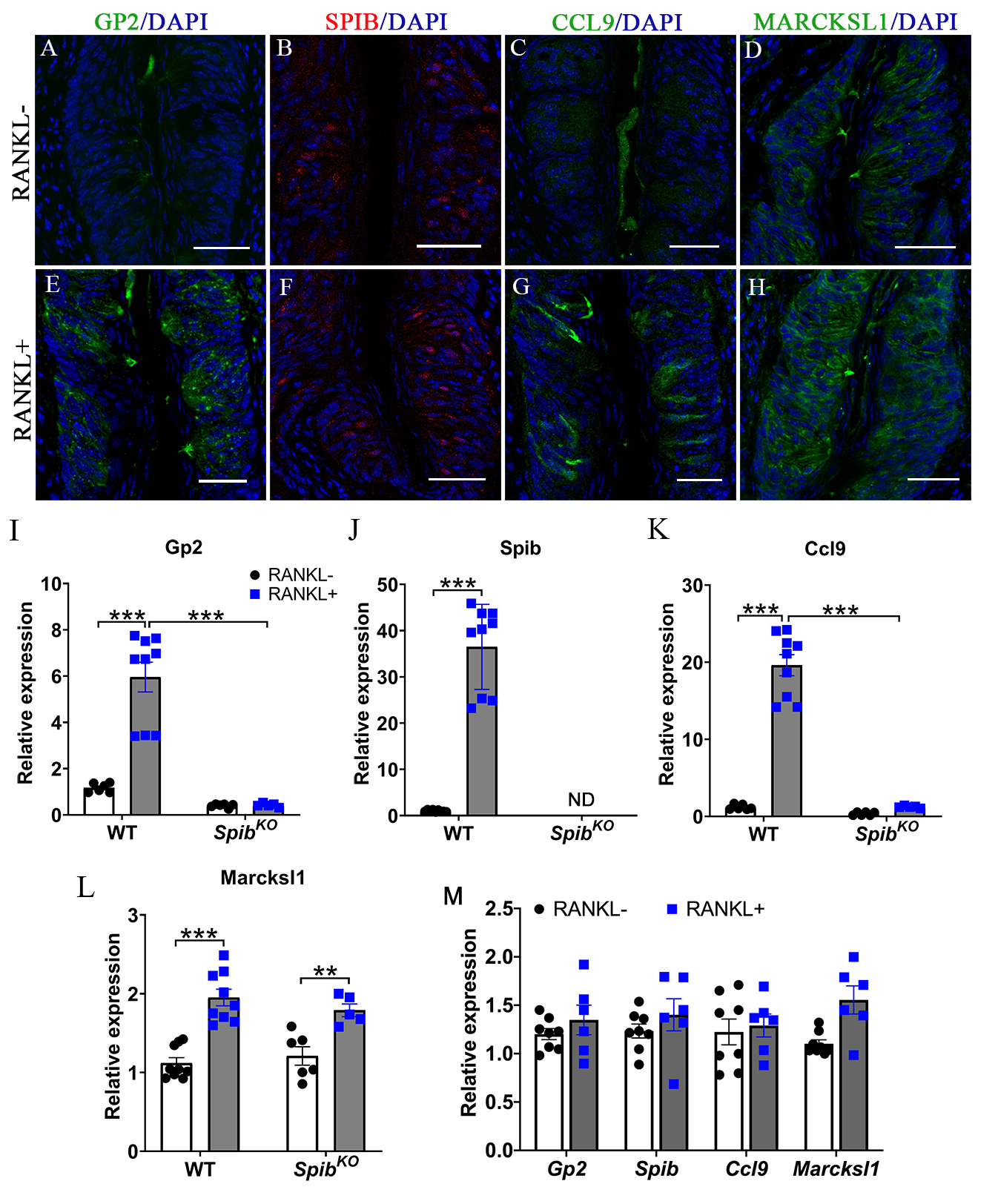

Supplement: S4 Fig — (A-H) Indirect immunofluorescence confocal microscopy of CVP sections from WT mice treated or untreated using RANKL stained with antibodies against M cell markers GP2, SPIB, CCL9, and MARCKSL1. The result showed that administration of RANKL led to a dramatic increase in the proportion of taste cells expressing these proteins. (I-K) qPCR analysis showed that all the above marker genes and Spib were significantly up-regulated after RANKL treatment in WT but not SpibKO mice. ND, not done. (M) RANKL treatment did not affect the expression level of M cell marker genes in NT lingual epithelium. Data are means ± SEM. (The data underlying the graphs can be found in Data I-M in S2_Data.) **p < .01, ***p < .001. Nuclei are counterstained blue with DAPI. Scale bar, 50 μm. (TIF) [file pbio.3001647.s006.tif]

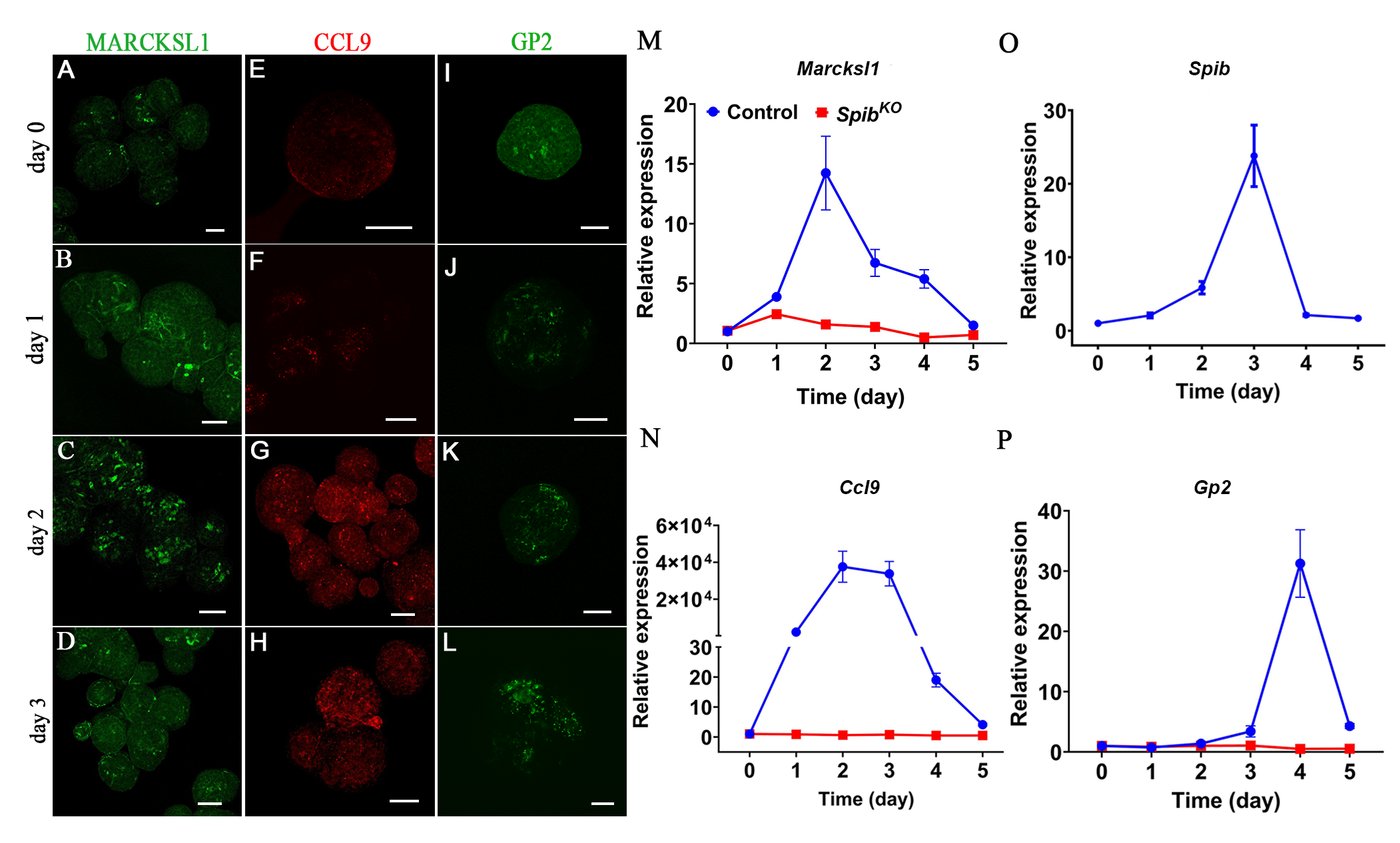

Supplement: S5 Fig — (A-L) Indirect immunofluorescence confocal microscopy of taste organoids from control and SpibKO mice showing kinetics of expression of the M cell markers MARCKSL1 (A-D), CCL9 (E-H), and GP2 (I-L) 0–3 days after RANKL treatment. (M-P) qPCR analysis of the expression of M cell markers in cultured taste organoids after RANKL treatment. The kinetics of expression of GP2, CCL9, and MARCKSL1 after RANKL treatment were distinct at both protein (A-L) and mRNA (M-P) levels. SpibKO mice failed to up-regulate all M cell marker genes (M-P) upon RANKL administration. (The data underlying the graphs can be found in Data M-P in S3_Data.) Data are mean means ± SEM. Scale bars, 50 μm. (TIF) [file pbio.3001647.s007.tif]

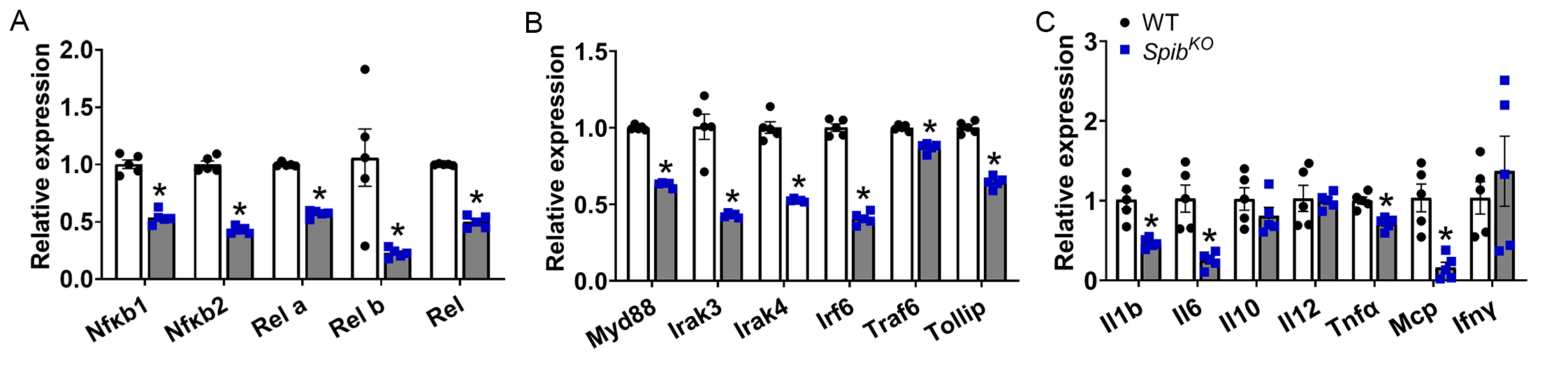

Supplement: S6 Fig — qPCR analysis of proinflammatory cytokines and the components of NF-κB signaling pathway in WT and SpibKO mice. (A-B) The expression of the transcription factors Nfkb1, Nfkb2, Rela, Relb, Rel, Irf6, and regulator proteins Tollip, Myd88, Irak3, and Irak4 belonging to the NF-κB signaling pathway and Irf6 are significantly down-regulated in SpibKO mice. (C) Compared to WT mice, SpibKO mice showed lower expression of proinflammatory cytokines Il1b, Il6, Tnf, and Mcp, but the expression of anti-inflammatory cytokines Il10 and Il12 did not change. (The data underlying the graphs can be found in Data A-C in S4_Data.) Data are mean means ± SEM. *p < .05. (TIF) [file pbio.3001647.s008.tif]

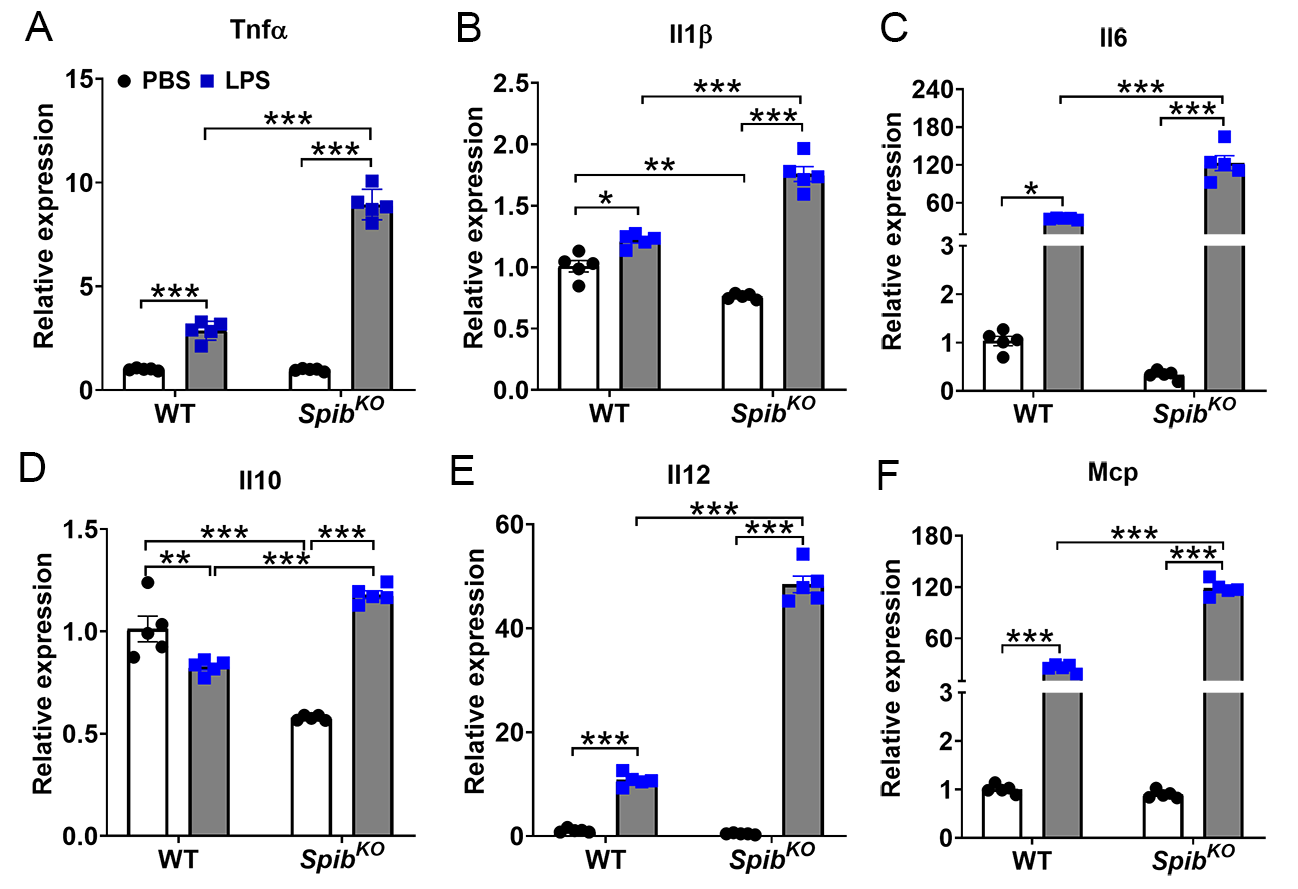

Supplement: S7 Fig — Compared to WT mice, LPS administration triggered exaggerated cytokine expression in CVP of SpibKO mice. (The data underlying the graphs can be found in Data A-F in S5_Data.) Data are means ± SEM. *p < 0.05, **p < .01, ***p < .001. (TIF) [file pbio.3001647.s009.tif]

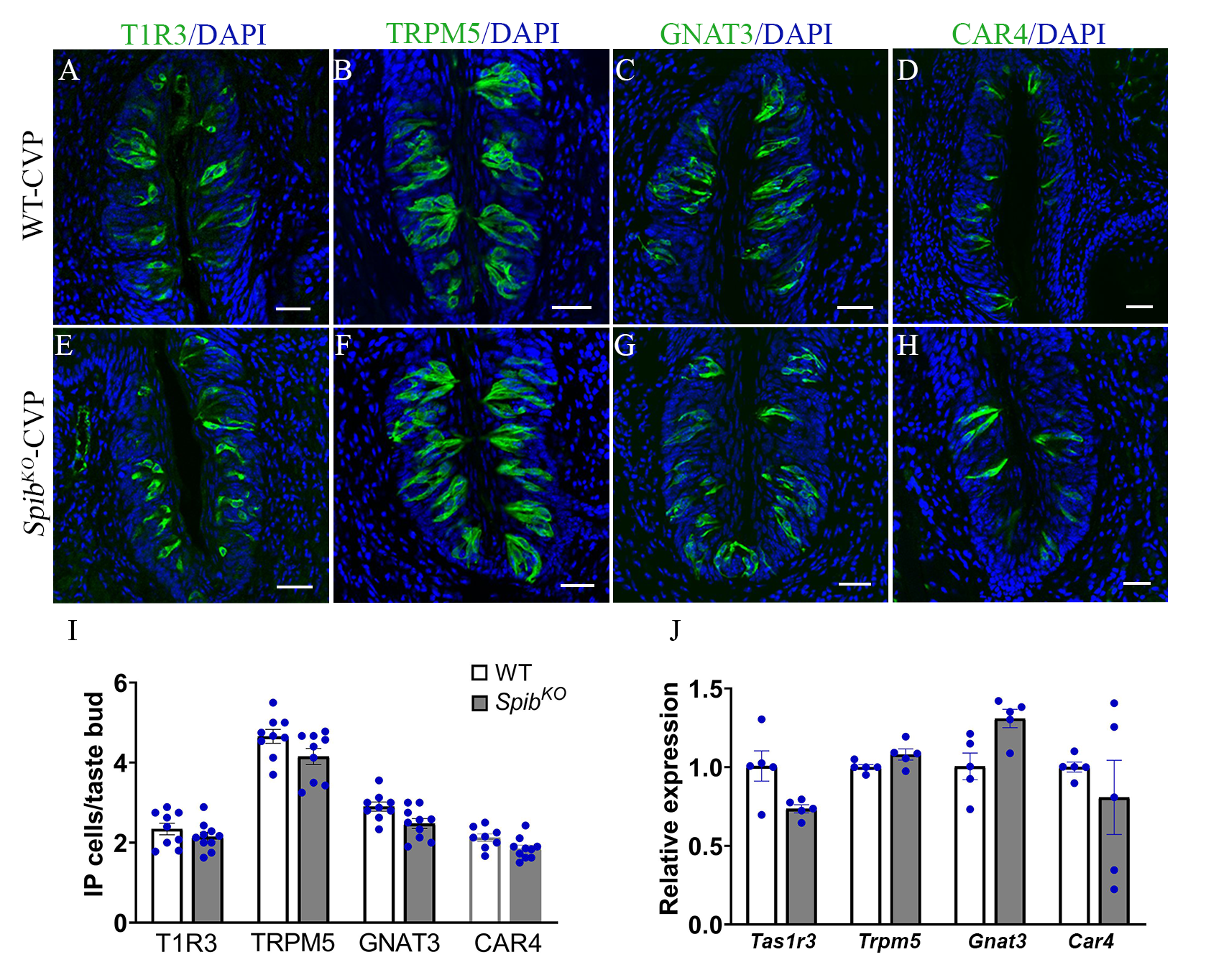

Supplement: S8 Fig — (A-H) Indirect immunofluorescence confocal microscopy of CV sections from WT and SpibKO mice immunostained for type II cells using markers T1R3 (A, E), TRPM5 (B, F), GNAT3 (C, G), and type III taste cells with antibodies against CAR4 (D, H). Nuclei are counterstained with DAPI (blue). (I) Compared to WT mice, the proportion of taste receptor cells in SpibKO mice were unaltered. (The data underlying the graphs can be found in Data I in S7_Data.) (J) qPCR analysis of expression of the corresponding genes confirms this observation (Data J in S7_Data). Data are mean means ± SEM with individual data points from replicates shown as black/blue dots. Scale bar, 50 μm. (TIF) [file pbio.3001647.s010.tif]

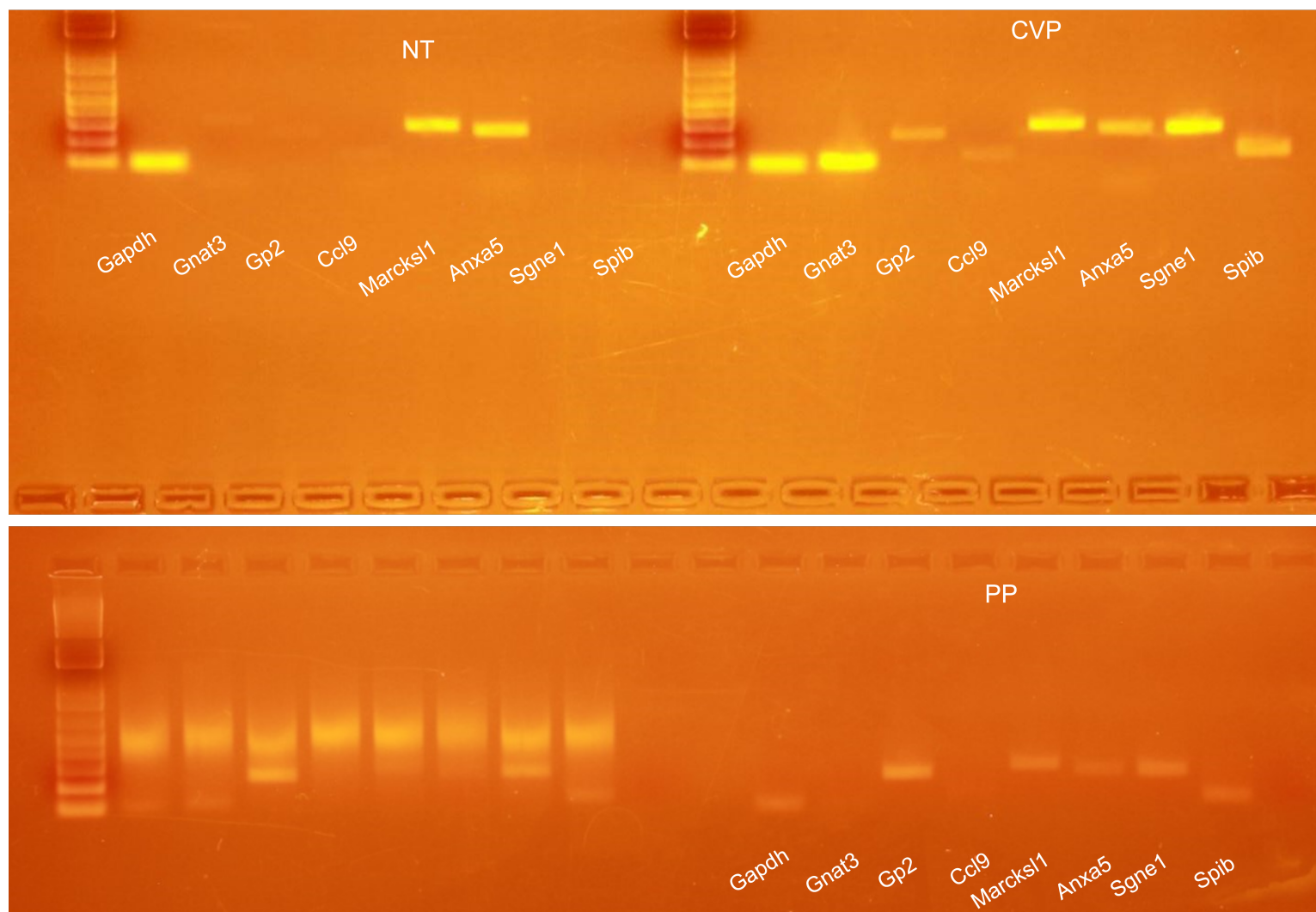

Supplement: S1 Raw Image — (PDF) [file pbio.3001647.s016.pdf]
