# Supplementary material for: Type II taste cells participate in mucosal immune surveillance
Source: PLoS Biol. 2023 Jan 12;21(1):e3001647. doi: 10.1371/journal.pbio.3001647 (PMC9836272; doi:10.1371/journal.pbio.3001647)
Supplement: S2 Table — RNAscope Hiplex assay was done in CVP with probe sets against Spib, Gp2, Tnfrsf11a, Tas1r3, Gnat3, Trpm5, and Car4, and singly and doubly labeled cells were counted. Numerators are the numbers of taste cells expressing both gene 1 and gene 2. Denominators are the numbers of taste cells expressing gene 1. Taste cells expressing both gene 1 and gene 2 as a percentage of those expressing gene 1 are shown in parentheses. ND, not determined. (DOCX) [file pbio.3001647.s012.docx]

| **Gene 1** | **Gene 2** | | | | | | |
| --- | --- | --- | --- | --- | --- | --- | --- |
|  | ***Spib*** | ***Gp2*** | ***Tnfrsf11a*** | ***Tas1r3*** | ***Gnat3*** | ***Trpm5*** | ***Ddc*** |
| ***Spib*** | - | ND | ND | 48/51 (94.1%) | 16/60 (26.7%) | 52/56 (92.8%) | 6/34 (17.6%) |
| ***Gp2*** | ND | - | ND | 24/47 (51.1%) | 42/56  (75.9%) | 21/22 (95.5%) | 8/45 (17.8%) |
| ***Tnfrsf11a*** | ND | ND | - | 21/50 (42.0%) | 10/23 (43.5%) | 39/45 (86.7%) | 12/26 (46.2%) |
| ***Tas1r3*** | 48/54 (88.9%) | 32/73 (43.8%) | 26/69 (37.7%) | ND | ND | ND | ND |
| ***Gnat3*** | 15/68 (22.1%) | 50/69 (72.5%) | 10/55 (18.2%) | ND | ND | ND | ND |
| ***Trpm5*** | 28/64 (43.8%) | 21/64 (32.8%) | 27/72 (37.5%) | ND | ND | ND | ND |
| ***Ddc*** | 9/63 (14.3%) | 7/52 (13.4%) | 9/33 (27.3%) | ND | ND | ND | ND |
