# Supplementary material for: Type II taste cells participate in mucosal immune surveillance
Source: PLoS Biol. 2023 Jan 12;21(1):e3001647. doi: 10.1371/journal.pbio.3001647 (PMC9836272; doi:10.1371/journal.pbio.3001647)
Supplement: S3 Table — Taste cells from CVP and FOP were doubly stained for SPIB and a second antibody against T1R3, GNAT3, TRPM5, or CAR4, and singly and doubly labeled cells were counted. Numerators are the numbers of taste cells expressing both gene 1 and gene 2. Denominators are the numbers of taste cells expressing gene 1. Taste cells expressing both gene 1 and gene 2 as a percentage of those expressing gene 1 are shown in parentheses. ND, not determined. (DOCX) [file pbio.3001647.s013.docx]

| **Gene 1** | **Gene 2** | | | | |
| --- | --- | --- | --- | --- | --- |
|  | **SPIB** | **T1R3** | **GNAT3** | **TRPM5** | **CAR4** |
| Nos. of circumvallate taste cells expression one or both genes | | | | | |
| **SPIB** | ̶ | 85/88 (96.6%) | 30/107 (28.0%) | 108/113 (95.6%) | 6/99 (6.1%) |
| **T1R3** | 85/91 (93.4%) | ̶ | ND | ND | ND |
| **GNAT3** | 30/128 (23.4%) | ND | ̶ | ND | ND |
| **TRPM5** | 108/261 (41.4%) | ND | ND | ̶ | ND |
| **CAR4** | 9/120 (5.0%) | ND | ND | ND | ̶ |
| Nos. of foliate taste cells expression one or both genes | | | | | |
| **SPIB** | ̶ | 41/45 (91.1%) | 5/43 (11.6%) | 21/23 (91.3%) | 2/51 (3.9%) |
| **T1R3** | 41/43 (95.3%) | ̶ | ND | ND | ND |
| **GNAT3** | 5/66 (7.6%) | ND | ̶ | ND | ND |
| **TRPM5** | 21/40 (52.5%) | ND | ND | ̶ | ND |
| **CAR4** | 2/45 (4.4%) | ND | ND | ND | ̶ |
