# Supplementary material for: Type II taste cells participate in mucosal immune surveillance
Source: PLoS Biol. 2023 Jan 12;21(1):e3001647. doi: 10.1371/journal.pbio.3001647 (PMC9836272; doi:10.1371/journal.pbio.3001647)
Supplement: S4 Table — (DOCX) [file pbio.3001647.s014.docx]

| **Gene** | **Forward primer** | **Reverse primer** |
| --- | --- | --- |
| *Gapdh* | GCATGGCCTTCCGTGTTCCTA | GATGCCTGCTTCACCACCTTCT |
| *Gnat3* | CATGGCTACACTGGGGATTG | GATTTCAGCCAGCTGTGGAG |
| *Gp2* | CCTGCGTTCTGACACTG | GCCGTGCAGGTTATCA |
| *Ccl9* | TACTGCCCTCTCCTTCCTCA | TTGAAAGCCCATGTGAAACA |
| *Marcksl1* | TTTTGCCCTCCTGTGGATTCT | CCACTAGGCACAGCACAAGAGA |
| *Anxa5* | AGGGCTGATGCAGAAGT | TCCCTGCCAAACACAG |
| *Sgne1* | ACGGTTAAAAATGGCCTCAAGG | AAGGACCCAGATGCTGAAGACC |
| *Spib* | GGGGGCCTTGACTCTA | CTCTGGGGGGTACACC |
| *T1r2* | GTCATTGGCCCAGACAACTC | TCGGTGATGGCGCTATATGT |
| *T1r3* | CAAGTTCTTCAGCTTCTTCC | GGCGGCCACCCAGTTCCAGC |
| *Car4* | CAATGGGTCAATGTGCTCTG | GGGGACTGCTGATTCTCCTT |
| *Il1b* | GTAATGAAAGACGGCACACC | ATTAGAAACAGTCCAGCCCA |
| *Il6* | TCATATCTTCAACCAAGAGGTA | CAGTGAGGAATGTCCACAAACTG |
| *Il10* | AAGGCAGTGGAGCAGGTGAA | CCAGCAGACTCAATACACAC |
| *Il12* | CGAATCCAGCGCAAGAAAGA | GGAACACATGCCCACTTGCT |
| *Tnf* | CTTCTCATTCCTGCTTGTGG | ATCTGAGTGTGAGGGTCTGG |
| *Mcp* | CAGCAGGTGTCCCAAAGAAG | GACCTTAGGGCAGATGCAGT |
| *Ifng* | AGCAACAGCAAGGCGAAAA | CTGGACCTGTGGGTTGTTGA |
| *Nfkb1* | GCTGAGTCCTGCTCCTTCTAAA | CCTCTGTGTAGCCCAATCTGTTGC |
| *Nfkb2* | CGGTGGAGACGAAGTTTATTTGCTC | ATCTTGTGATAGGGCGGTGT |
| *Rela* | CGGGATGGCTACTATGAGGCTGAC | GGGTTATTGTTGGTCTGGATTCGC |
| *Relb* | ACACCCACATAGCCTCGT | ATTTCCTTCTTCCTAACACACTGGA |
| *Rel* | ACCCAATTTATGACAACCGTGCCC | ACCTCTGGCTTCCCAGTCCATTCAA |
| *Myd88* | TAAGTTGTGTGTGTCCGACCGTGA | ATCAGTCGCTTCTGTTGGACACCT |
| *Irak3* | AGAGCTGGCTGCATATTTCACGGA | GGGTTGTGCCATTTGTGCACTGTA |
| *Irak4* | ACGGGCTTCGGCAAGGCTA | ATCCAGCAGTAGTTGAGGTTCACG |
| *Irf6* | AGAGGAACCAGAGATTGACAGC | GTCAGAGACCCAAACTATGGTGC |
| *Traf6* | TTTACTGGGAAGCAGTGCAAACACC | ATTTGGGCACTTTACCGTCAGGGA |
| *Tollip* | GGTCGCCTCAGCATCACT | TCATAAACAGCATAGCCCAGACGCA |
