# Supplementary material for: Type II taste cells participate in mucosal immune surveillance
Source: PLoS Biol. 2023 Jan 12;21(1):e3001647. doi: 10.1371/journal.pbio.3001647 (PMC9836272; doi:10.1371/journal.pbio.3001647)
Supplement: S5 Table — *RRID, Research Resource Identifier. (DOCX) [file pbio.3001647.s015.docx]

| **Antibody** | **Host** | **Cat. no.** | **RRID*** | **Source** | **Dilution** |
| --- | --- | --- | --- | --- | --- |
| CAR4 | Goat | AF2414 | AB_2070332 | R&D Systems, Minneapolis, MN | 1:50 |
| TRPM5 | Guinea pig | － | － | Dr. Emily Liman, University of Southern California, Los Angeles | 1:800 |
| ENTPD2 | Rabbit | － | － | Dr. J. Sevigny, Laval University, Quebec, Canada | 1:250 |
| GP2 | Mouse | D278-3 | － | MBL, Nagano, Japan | 1:200 |
| CCL9 | Goat |  | － | R&D system, Minneapolis, Minn, USA | 1:100 |
| MARCKSL1 | Rabbit | 11422-1-AP |  | Proteintech, Rosemont, USA | 1:300 |
| SPIB | Rat |  | － | Dr. Lynn Corcoran, Walter and Eliza Hall Institute for Medical Research, Australia | 1:500 |
| CD45 | Rat | 550539 | － | BD Pharmingen, Becton, USA | 1:500 |
| CD3 | Rat | 555273 | － | BD Pharmingen, Becton, USA | 1:500 |
| CD11B | Rat | 550282 | － | BD Pharmingen, Becton, USA | 1:500 |
| Anti-rabbit IgG-Alexa 488 | Donkey | A11008 | － | Invitrogen, Eugene, OR | 1:1000 |
| Anti-rabbit IgG-Alexa 555 | Donkey | A31572 | － | Invitrogen, Eugene, OR | 1:1000 |
| Anti-rabbit IgG-Alexa 647 | Donkey | A31573 | － | Invitrogen, Eugene, OR | 1:1000 |
| Anti-goat IgG-Alexa 488 | Donkey | A21206 | － | Invitrogen, Eugene, OR | 1:1000 |
| Anti-goat IgG-Alexa 555 | Donkey | A21432 | － | Invitrogen, Eugene, OR | 1:1000 |
| Anti-guinea pig IgG-Alexa 647 | Donkey | 706-605-148 | － | Thermo Fisher, Carlsbad, CA | 1:1000 |
