# Supplementary material for: Type II taste cells participate in mucosal immune surveillance
Source: PLoS Biol. 2023 Jan 12;21(1):e3001647. doi: 10.1371/journal.pbio.3001647 (PMC9836272; doi:10.1371/journal.pbio.3001647)
Supplement: Attachment — Submitted filename: Response to reviewers comments.docx [file pbio.3001647.s017.docx]

**Response to reviewers’ comments:**

The authors wish to thank the reviewers or their insightful comments and criticisms. Below, please find a point-by-point response to each reviewer.

**Response to reviewer 1:**
These authors demonstrate that taste receptor cells express key genes and proteins involved in M cell surveillance of the gut microbiome and might play a similar role in the oral cavity. This is an exciting study that opens the door to novel taste cell-microbial interactions. The work is also timely given the altered oral microbiome in COVID-19 patients and a possible link to taste loss. The authors used compelling mix of in vivo and organoid models. The manuscript is generally well-written and most of my suggestions are relatively minor. Most significant are the lengthy presentation of results in the introduction and questions about interpreting results.

* In the title and throughout the manuscript the focus is on sweet taste receptor cells. While that might be shorthand for sweet- and umami-sensing type II cells I don't find "STRC" accurate given expression and behavioral data.

*We agree that multiple taste cell types other than STRCs may participate in immune surveillance. At the same time, type II taste cells seem to have a special role in this, in view of the fact that Pou2f3 knockout mice do not express M cell marker genes in CVP (figure S1). Thus, we have changed our title and have also replaced STRCs with Tas1r3+ cells in the text.*

* The introduction would be improved by summarizing the main findings more concisely.

*We have shortened the description of results in the introduction section.*

* Methods
1) Are mice housed in a SPF barrier facility which relates to microbial exposure?

*Yes, the mice were housed in an SPF barrier facility (please see line 404 in text).*

2) What is the route and dose of LPS administration?

*The following text was added to the methods section to describe the route of LPS administration. ‘Half of WT and Spib^KO^ mice received a single intraperitoneal injection of 5 mg/kg LPS (dissolved in PBS), and the other half received an injection of vehicle (PBS buffer) as control’. (Line 433- 35)*

3) Data were analyzed by two-way ANOVAs followed by t tests as multiple comparisons. Were P values adjusted to control Type I error introduced by multiple t tests?

*During data analysis of behavioral experiments, Bonferroni correction was applied to avoid potential false-positives owing to multiple comparisons. The level of statistical significance was defined as P<0.05/4=0.0125. (Line 576-77).*

4) Provide details for confocal imaging including objective, zoom and optical thickness of z scans.

*The requested information was added (Line 522-32).*

* Figure 1L. What are the heavily stained SPIB+ cells at the base of foliate papillae? If they are glands this might be discussed later in comparison to the gut.

*These do not have the appearance of salivary glands. We think it likely that they are immune cells such as dendritic cells. However, we have not tested this by staining with immune cell markers so have not discussed their nature.*

* Figure 2. The two lower magnification panels make it difficult to see co-localization vs. background.

*We agree that the background is higher than preferred. However, we were unable to repeat these IHC experiments since both primary authors have left Monell center where the bulk of this work was done and we no longer have the SPIB antibody available to us.*

* Page 8 line 181. The statement that "Collectively, the results above conclusively show that STRCs express several M cell marker genes" in not precise. Some of the genes were co-localized with TRPM5/type II markers rather than T1R3.

*Agreed. With this in mind and as mentioned above, we have changed our title and text to reflect the fact that type II and even type III cells may mediate immune surveillance.*

* Figure S2 and accompanying text/legend. The results demonstrate an increase in M cell marker genes upon treatment with RANKL but not proliferation, which was not directly tested.

*Agreed. We have edited the text to remove mention of cell proliferation and changed it to changes in the proportion of cell types. (Lines 212- 14 and 222 -25).*

* Legends for Fig. S3 and 5 have the same title. It was also not clear why some of the results were presented as supplementary figures rather than included in the main text unless there are journal limits.

*We agree that more data can be moved to the main text, and have moved figure S5 to the main text (now Figure 7).*

* Figure 5. The images showing bead uptake are not very convincing. Were beads at the apical surface of the taste counted? It appears that only 2-3 beads are present within the taste bud.

*We have replaced the images showing bead uptake (Figure 6). Since we are no longer focusing only on STRCs, we have stained with KCNQ1 to visualize all taste cells.*

* To my knowledge, M cells fail to develop from progenitors in SPIB KO mice. In contrast there were no differences in the different types of taste cells in KO mice. The difference in the gut and taste cell phenotypes in SPIB KO mice is striking and could be added to the discussion section comparing and contrasting taste cells and M cells.

*We have edited text in the discussion section to highlight this (Lines 388-91).*


**Response to reviewer 2:**
This manuscript reports that the subset of taste receptor cells that respond to sweet tastes express markers for M cells of the immune system, including SPIB. A comparison between the taste cells and immune cells is further supported by experiments showing that administration of RANKL leads to an increase in the number of cells that express these markers, while expression of these markers is lower in a Spibko mouse strain. Most remarkably, the authors find that Spibko mice show a heightened preference for sucrose and MSG over water, as compared to WT mice. Overall, there are some interesting and potentially very important findings in this manuscript.

Major:

1. Much of the paper rests on immunocytochemistry to define the population of cells that express SPIB. However, because SPIB is nuclear, and the taste cell markers are on the plasma membrane (or cytosol), it is sometimes difficult to tell in which cells they are co-expressed (and how this was assessed by the authors). This is made more difficult by quality of some of the images. One solution would be to use the TRPM5-GFP mouse strain generated by this PI, which should show better colocalization. It would also be helpful if the authors added a Venn diagram to show cell counts (cells in which the proteins are co-expressed, or expressed alone).

*Due to the difficulty with accurately determining colocalization based on IHC data for the nuclear SPIB and the microvillar GP2, we have added RNAscope Hiplex data showing colocalization between M cell and taste cell marker genes. Findings from this assay generally agree with those from IHC (Figure 2 and Table S2).*

2. Organoid IHC is not convincing given low-resolution images and poor morphology of the cells, and adds little to the manuscript. It could be put in a supplementary figure.

*We have moved the organoid IHC data to a supplementary figure (Figure S2) and moved the data from RANKL treated CVP to the main text.*

3. Images showing bead uptake are not convincing - the beads appear to be stuck at what appears to be the apical surface of the tastebud (labeling the taste pore would be helpful). The negative control does not have a similar number of T1R3 labelled cells. Did the investigators ever observe beads inside the cells? This should be shown if possible.

*We agree that a lot of beads are stuck at the trench region of CVP, perhaps bound to mucins. We have now provided new images in which taste cells are counterstained with KCNQ1 (Figure 6 G-H). Spib knockout mice clearly show inability to take up beads, but it is difficult to determine which cell types are more proficient at taking up beads, as beads may be transcytosed out of cells that take them up from the lumen during our assay.*

4. The increased preference for sucrose and MPG is impressive. But some critical questions need to be answered. First, it is surprising that a similar change in preference is not seen for sucralose. How can this be explained? And without a mechanism, this change in behavior could be due to global effects of the knockout (on M cells throughout the body, leading, for example, to changes in metabolism). To show that there is a change in the peripheral response of taste cells, and connect the results to the main findings of the papers, the authors should, at a minimum, show results from gustatory nerve recording.

*We agree that obtaining nerve recording data would be desirable. Unfortunately, both primary authors have left the Monell Center and the Spib knockout mice strain is no longer maintained in any of our mice colonies. Hence, we are unable to generate this data. With regard to differences between preference for sucrose and sucralose we speculate that it may have to do with caloric responses to sucrose via the so-called taste metabolic pathway we have previously reported (PMID: 21383163,* *PMID: 27162343).*

5. Is there something about this strain or the way that the animals were tested that makes them so insensitive to ionic tastes? First, the mice continue to lick high concentrations of NaCl (300 mM and 600 mM) that are typically very aversive (at 600 mM, the still lick at >50% of the rate that they lick to H2O). Also, concentrations of citric acid that are typically strongly aversive (30 mM - 300 mM) appear to not be aversive or only mildly aversive, and the concentrations in the figure are different from those in the methods.

*Apologies for generating confusion on this point. We made a mistake with the concentrations while plotting the graph for behavioral response to citric acid. We have edited this in Figure 8 D to reflect the correct range of citric acid used (1-30 mM). We are not sure why the aversion to NaCl is not as strong as we usually see in behavioral assays, but this appears to not to be related to Spib ablation, as the WT mice also show a similar behavioral response to NaCl. We typically see very strong aversion at 600mM – 1M NaCl, although this was not tested in this study.*

Minor:
Fig 1 A - please include MW markers

*We have added the position of MW markers.*

Fig 1B - the gene "Spi-B" is presumably Spib?

*We have edited the typo for Spib.*

Fig 1C-J - label figure with name of probe Fig K-N - In the absence of taste cell markers, it is hard to tell where the taste buds are in the IHC, making the expression of SPIB not very convincing. Same for KO. I suggest leaving this out, and simply showing the IHC in Fig 2, along with the KO controls (counterstained for taste cell markers).

*We have edited Figure 1, and have replaced in situ hybridization images with RNAscope images.*

Fig 6 A - SpiB knockout - should be Spib? (gene name)

*Yes, this is now corrected.*

Methods: Was tissue fixed in PFA for 1h or overnight (line 452)?

*The tissues were fixed in PFA for one hour (Line 484).*


**Response to reviewer 3:**
In this manuscript the authors suggest that a subset of sweet sensitive taste receptor cells in mouse taste buds functions in immune surveillance of pathogens in the oral cavity. The mechanistic hypothesis is very exciting and very timely given the impact of SARS-CoV2 on smell and taste function. They interrogate their published scRNAseq dataset of 3 different taste bud cell types for genes expressed by microfold (M) cells that function in other immune tissues and show that several of these are expressed in taste cells. They first use immunostaining to show several microfold cell marker proteins, including Spib, Gp2, and Ccl9, are co-expressed in taste bud cells. They then use organoid technology to activate the immune response with the RANKL, the ligand for Tnfrsf11a, which they find enriched in taste bud cells in the RNAseq dataset, and show substantial upregulation of several M cell gene products in response to RANKL. They further show that Spib is required for this
response in organoids and in mice. In vivo, M cells have epithelial transcytosis function and can take up fluorescent beads, and the authors show data that Tas1r3+ cells in mouse taste buds also take up these beads and uptake is Spib-dependent. Finally, they use a behavioral assay to show that compared to controls, Spib-cKO mice have altered taste responses to sweet and umami tastants, but not to bitter, salt or sour, and suggest that in addition to functioning in immune surveillance, sweet taste cells might "might modulate taste preference in response to microbial colonization and infection "

While these are intriguing findings, their data do not appear to fully support a model where sweet taste receptor cells uniquely function to mediate oral immune responses. In fact their data suggest that there are complex expression patterns of M cell genes in taste epithelium and complex responses to immune challenge in taste epithelium that are not consistent with their interpretation.

The authors rely on their published scRNAseq dataset of mouse CVP taste cells, made up of only 32 cells and roughly 10 cells per cell type (GAD1+ type III, Gnat3+ bitter type II and Tas1r3+ sweet and umami type II taste cells). In table S1, the authors present M cell genes enriched in their dataset. Combined with the highly variable expression of each gene within each group, comparisons of differentially regulated genes across groups should be more cautiously interpreted by the authors here. Comparing Tas1r3+ sweet and umami type II cells vs Gad1+ type III cells for example show differences between these 2 populations, however, the authors are making the strong statement that these immune related genes are upregulated specifically in sweet taste receptor cells. Of interest, some genes they suggest are functionally involved in immune response of taste buds are actually most highly expressed in Gad1+ cells, i.e., Gp2, Tnfrsf11a. They include scRNAseq expression data also for
the Gnat3+ bitter type II cells. A quick comparison of Gnat3+ and Tas1r3+ cells shows that many of these genes are not upregulated specifically in sweet cells but instead are expressed in both type II populations, e.g. Spib, or more highly, although quite variably, expressed in bitter cells e.g., Scg5, Gp2. PCR and immunofluorescence analysis (Figs 1-3) also suggests that these M cell genes are expressed in a complex pattern among bitter and sweet/umami cells, as well as in the non-taste epithelium of the tongue (Marcksl1, Anxa5). The authors appear to gloss over this complexity:"Using single cell RNASeq of GFP-labeled mouse taste cells, we found that sweet taste receptor cells (STRCs) and a few other taste cell types express several M cell marker genes" It is also important to note that Tas1r3 is also expressed by umami-sensitive taste buds cells, and thus limiting the interpretation of the function of Tas1r3+ cells to sweet taste cells is an oversimplification as well.

*We fully agree with these comments, and our previous data and the newly generated RNAScope data show that cells other than STRCs are likely involved in immune surveillance. However, type II cells appear to have a special role at least in the basal state, as the expression of M cell marker proteins is abrogated in Pou2f3^KO^ mice (Figure S1). Hence, we have made changes to the title and throughout the text to reflect this point.*

Additional experiments that would improve the manuscript would be to show explicit co-expression of M cell genes with Tas1r2 mRNA in taste tissue.

*We no longer posit that STRCs are the primary cell type involved in immune surveillance, making this experiment moot.*

Specific critiques
Immunofluorescence for GP2 or CCL9 in Fig 3 appear to show many cells in taste buds express these proteins, not just TRPM5+ taste cells, which is expressed by bitter, sweet and umami type II cells. In panel 3D-F, GP2 appears to also be expressed in a CAR4+ cell, and the extent to which GP2 is co-expressed in 3A-C is difficult to determine as immunofluorescence is brightest at the taste pore. Given the apparent high background of GP2 and CCL9 the authors should show control tissue with primary antisera omitted, especially since GP2 is a mouse antibody.
Further in Fig S2, GP2 appears not to be expressed in control taste tissue, only in that from mice treated with RANKL, and with RANKL treatment many more cells appear immunopositive for GP2, CCL9 and MARCKSL1, and SPIB than in controls and that can be accounted for by expression specific to only Tas1r3+ sweet/umami cells. The authors should assess these expression patterns with TAS1R3 double labeling.

*We agree that the IHC data by itself is not conclusive. Hence, we have added RNAScope data showing the coexpression of M cell marker genes with taste marker genes. Since we do not have Spib^KO^ mice in our animal colony, we could not compare the expression of M cell markers in RANKL treated Spib^KO^ vs. control (WT) mice.*

In Figs 2, 3 and 5 the authors do not indicate if these are projections of confocal z-stacks or optical sections; the latter are required to determine if a single taste cell is double labeled, or if a single cell has taken up a fluorescent bead. In figure 5, how many taste buds and cells were analyzed for bead transcytosis?

*We have added the information of confocal imaging, see line 522-32. The cumulative thickness of z-stacks was maintained below 6 µm (the average thickness of taste cells) to avoid false colocalization.*

*In bead transcytosis assay, only the fluorescent beads on CVP area (as shown in the following figure, the yellow dotted line marks area) were analyzed.* *Two or three sections with entire CVP of each mouse was counted for bead transcytosis assays (N=5).*

The authors should show individual datapoints for plots in Fig 1B, 4M-P, 5I, and 6A-F, S2I-L, S3A-C, S4A-F, S5B-F, .

*We have edited all these figures to show individual datapoints.*

In table S2, how many mice were used in this analysis?

*We have described the number of mice used (N= 3) in the text (Line 529-30).*

*Other changes: The new RNAScope experiments showed that Spib is also expressed in the fungiform papillae (Figure 1 D). Older results already show that it is expressed in circumvallate and foliate papillae.*
